# Effect of a multi-citrus extract-based feed additive on the survival of rainbow trout (*Oncorhynchus mykiss*) following challenge with *Lactococcus garvieae*

**DOI:** 10.1186/s13028-020-00536-0

**Published:** 2020-07-01

**Authors:** Brenda Mora-Sánchez, Héctor Fuertes, José Luis Balcázar, Tania Pérez-Sánchez

**Affiliations:** 1 Department of Animal Pathology, Faculty of Veterinary Sciences, Universidad de Zaragoza, Saragossa, 50013 Spain; 2 Department of Animal Health, Centro Veterinario de Diagnóstico e Investigación (CEVEDI), School of Veterinary Medicine, Universidad Nacional Autónoma de Nicaragua-León, León, Nicaragua; 3 Catalan Institute for Water Research (ICRA), Girona, 17003 Spain; 4 University of Girona, Girona, 17004 Spain

**Keywords:** Antibacterial activity, Biocitro^®^, Feed additive, Fish bacterial pathogens, Rainbow trout

## Abstract

Growing global concerns about antibiotic resistance have generated a considerable interest in the search for alternative environmental-friendly approaches. This study was aimed to assess the antimicrobial activity of a multi-citrus extract-based feed additive (Biocitro^®^) against some fish pathogens, as well as evaluate its capacity to protect rainbow trout (*Oncorhynchus mykiss*) to lactococcosis. A broth dilution method was used to determine the minimum inhibitory concentration (MIC) of Biocitro^®^, and the results showed a strong antibacterial activity against *Aeromonas salmonicida*, *Lactococcus garvieae* and *Yersinia ruckeri* with MIC values of 2.0 µg/mL. Afterwards, rainbow trout juveniles were fed a Biocitro^®^-enriched diet (750 mg/kg feed) at a daily rate of 1.5% body weight for 4 weeks, then they were challenged with *L. garvieae* by the cohabitation method. At the end of the experimental period, fish treated with Biocitro^®^ showed significantly (P < 0.001) improved protection against *L. garvieae* compared to control fish. Although further studies are needed to understand how Biocitro^®^ increases rainbow trout resistance to *L. garvieae*, this feed additive could be considered as a useful alternative to chemotherapeutic treatment in aquaculture.

## Findings

Growing global concerns about antibiotic resistance and limited efficacy of vaccination have prompted the search for alternative environmental-friendly approaches in aquaculture [1]. The use of plant-derived natural compounds (phytobiotics) is gaining a considerable interest as an alternative to antibiotics, which should be restricted to prevent the emergence and spread of antibiotic-resistant bacteria [1–3]. Biocitro^®^ (Quinabra; São José dos Campos, SP, Brazil) is a commercially available natural feed additive specifically designed for animal use. According to the manufacturer’s specification, the product contains a defined blend of citrus extracts including grapefruit (*Citrus paradisi*), tangerine (*Citrus reticulata*), bergamot (*Citrus aurantium* ssp. *bergamia*), and sweet orange (*Citrus sinensis*), whose active compounds are ascorbic acid, citrus bioflavonoids (hesperidin, naringin, quercetin and rutin), and organic acids [4]. The aim of this study was to evaluate the antimicrobial activity of Biocitro^®^ against some fish pathogens, as well as investigate its capacity to protect rainbow trout (*Oncorhynchus mykiss*) to *Lactococcus garvieae* infection.

The pathogens *Aeromonas salmonicida* (strain CLFP 30), *L. garvieae* (strain CLFP 33) and *Yersinia ruckeri* (strain C4R7), previously isolated from rainbow trout during natural outbreaks and identified by standard microbiological methods, were grown on tryptic soy agar (TSA; Oxoid, Basingstoke, UK) overnight at 22 ± 2 °C. Afterwards, bacteria were collected and resuspended in phosphate-buffered saline (PBS; pH 7.4). Bacterial suspensions were spectrophotometrically adjusted to an absorbance (600 nm) of 0.125 ± 0.05, that corresponded to a reference concentration of 10^7^ CFU/mL. Serial dilutions of each bacterial suspension were spread onto TSA duplicate plates and the number of colony-forming units (CFUs) was counted after incubation at 22 ± 2 °C, in order to verify the bacterial inoculum concentration. A broth dilution method was then used to determine the minimum inhibitory concentration (MIC) of Biocitro^®^ against the three pathogens, according to Clinical and Laboratory Standards Institute guidelines [5]. Briefly, a volume of 1.0 mL of Biocitro^®^, previously filtered, was added to sterile test tubes containing 1.0 mL of tryptic soy broth (TSB) and was serially two-fold diluted to obtain concentrations ranging from 0.25 to 250 µg/mL. The test tubes were then inoculated with 1.0 mL of fresh bacterial cultures at a final concentration of 10^5^ CFU/mL. All assays were carried out in triplicate, including also a negative control (with medium only) and a positive control (with the reference antibiotic, oxytetracycline). Moreover, tubes containing bacteria in TSB without any other substance were used to verify bacterial growth whereas a tube containing only TSB without bacteria was used to check medium sterility during the experiments. After 24 h incubation at 22 ± 2 °C, bacterial growth was examined by observing the turbidity of tubes. An absence of turbidity was interpreted as absence of growth whereas the presence of turbidity was interpreted as positive growth. The MIC was defined as the lowest concentration (µg/mL) of Biocitro^®^ or antibiotic that completely inhibited the visible growth of all the three bacterial species, when compared with the TSB control [6]. The results showed that 3.9, 7.8 and 7.8 µg/mL of oxytetracycline inhibited the growth of *A. salmonicida* strain CLFP 30, *L. garvieae* strain CLFP 33 and *Y. ruckeri* strain C4R7, respectively. Similar MIC values have been previously reported against other strains of these bacterial species isolated from farmed fish, shrimp, and their surrounding environment (water and sediment) [7–9]. The results also revealed that 2.0 µg/mL of Biocitro^®^ was enough for inhibiting all tested strains. Other authors have previously shown that citrus extracts possess a strong inhibitory activity against yeasts (*Saccharomyces bayanus*, *Pichia membranifaciens* and *Rhodotorula bacarum*) and bacteria (*Brachyspira hyodysenteriae*, *Lactobacillus plantarum*, *Lactobacillus brevis* and *Bacillus coagulans*) [4, 10], thus supporting our results.

To evaluate whether Biocitro^®^ confers to fish beneficial effects against bacterial infections, rainbow trout juveniles were fed a diet supplemented with Biocitro^®^ for 4 weeks and then they were challenged with *L. garvieae*. Briefly, a total of 100 pathogen-free rainbow trout were obtained from a commercial fish farm (Viveros del Soto Oliván) in the Autonomous Community of Aragon, Spain. After an acclimatization period of 2 weeks, fish (mean body weight 25.0 ± 5.0 g) were randomly assigned to three experimental groups and maintained in three tanks. Specifically, two groups were fed a commercial feed (Inicio Plus 887; BioMar Iberia, S.A., Dueñas, Spain) without any supplement: one group (*n *= 40) was used as untreated control and submitted to infection, whereas the other group (*n *= 20) was used for the experimental infection as donors. The third group (*n *= 40) received a diet obtained by adding Biocitro^®^ to the commercial diet at 750 mg/kg and then was infected. Water quality parameters were measured daily, and temperature (17.0 ± 0.8 °C), dissolved oxygen (6.7 ± 0.5 mg/L), pH (6.9 ± 0.2), nitrite (0.03 ± 0.01 mg/L), and nitrate (0.8 ± 0.2 mg/L) were constant throughout the experiment. All fish were maintained in aerated fresh water with a 25% water change every day and a 12 h dark/12 h light photoperiod, and they were fed daily at 1.5% of their biomass. After 4 weeks of feeding, fish were individually weighed to evaluate the effect of Biocitro^®^ on growth. Subsequently, fish were challenged with *L. garvieae* by cohabitation method [11, 12]. *L. garvieae* cells, previously grown on TSA plates, were collected and resuspended in PBS. Bacterial concentration was spectrophotometrically adjusted to 10^4^ CFU/mL, and serial dilutions were spread onto TSA plates to verify the infective dose. A volume of 0.1 mL was intraperitoneally injected into donors, which were previously anaesthetized with tricaine methanesulphonate (Tricaine Pharmaq 1000 mg/g) and marked by clipping the adipose fin. Then, ten infected fish were transferred into the tanks containing the other two experimental groups. All fish were monitored at least three times daily for 2 weeks, and dead fish were immediately removed and examined for external signs of lactococcosis, such as lethargy, irregular swimming behavior, exophthalmia and hemorrhages in the periorbital and intraocular area, perianal region and base of fins. Necropsy results indicated different severity of internal lesions, including enlarged spleen, extensive hemorrhages and ascites.

**Fig. 1:**
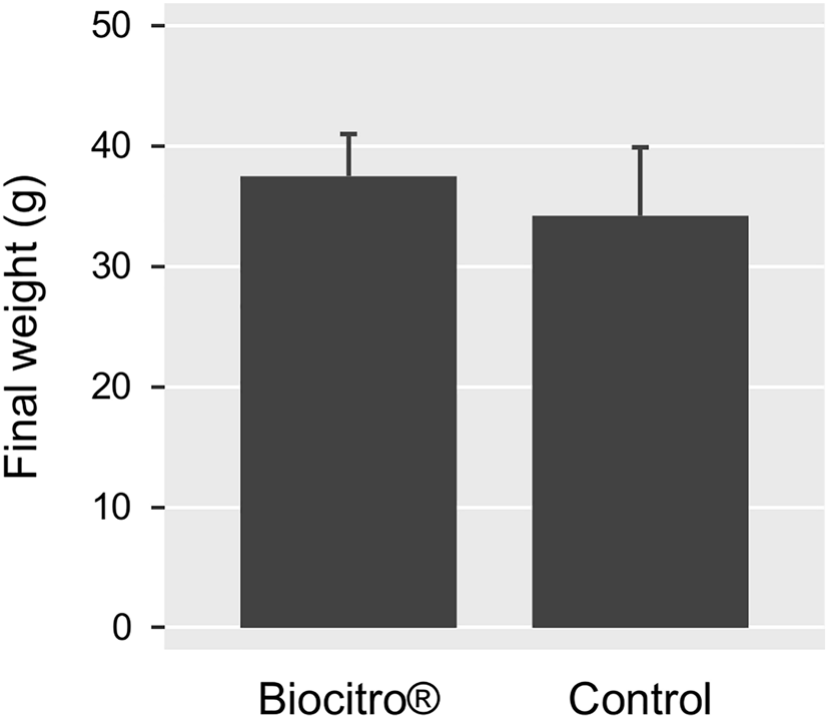
Final weight of rainbow trout fed the experimental diets for 4 weeks. Bars represent mean values and error bars represent standard deviations

**Fig. 2:**
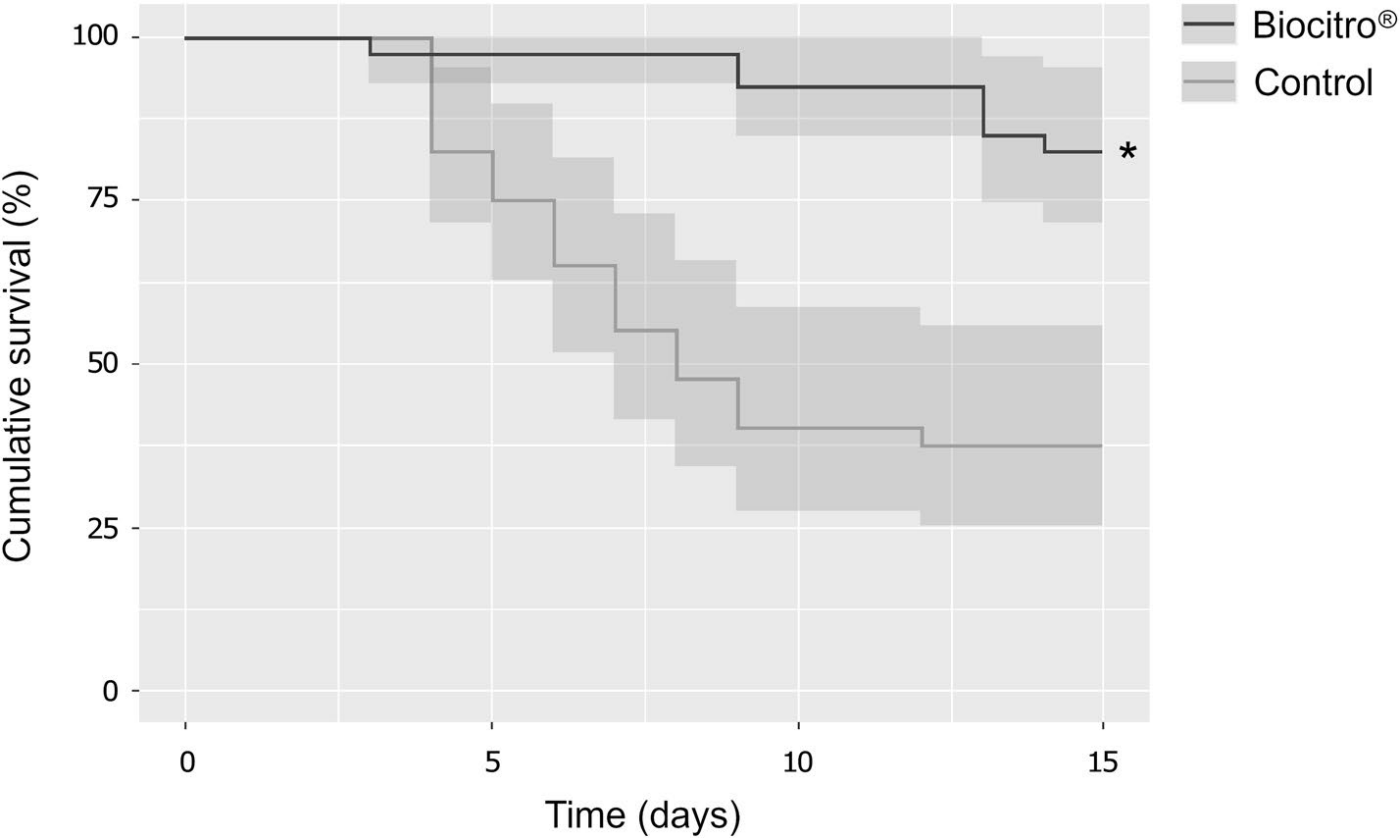
Cumulative survival of rainbow trout fed the experimental diets for 4weeks and challenged with *L. garvieae* by cohabitation. The asterisk indicates the significant difference (P < 0.001) between treated and control group

At the end of the experimental period, fish growth data were analyzed using unpaired two-tailed Student’s *t* test. Kaplan–Meier survival analysis was performed to investigate statistical differences among the experimental groups (P < 0.05). Statistical analyses were performed using R version 3.4.3 (R Foundation for Statistical Computing, Vienna, Austria). No significant difference (P = 0.30) was observed in the final weight between fish fed the Biocitro^®^-enriched diet and control group (Fig. 1). Moreover, no side effects associated with its use was observed. However, a significant difference (P < 0.001) in the cumulative survival between fish fed a Biocitro^®^-enriched diet and control group was recorded (Fig. 2). Cumulative survivals were 82.5% (95% CI 71.5–95.2%) and 37.5% (95% CI 25.1–55.9%) in treated and control groups, respectively. The relative percent survival (RPS) of treated fish was 72%, calculated as previously described [13]. These results seem to be consistent with previous studies, which revealed that the administration of phytobiotics in fish confers protection against bacterial infections. For instance, another commercially available phytogenic feed additive (Digestarom^®^) conferred protection against *A. salmonicida* to rainbow trout [14], and its dietary supplementation resulted also correlated with a statistically significant reduction of mortality in channel catfish (*Ictalurus punctatus*) challenged with *Edwardsiella ictaluri* [15]. These evidences suggest a direct antimicrobial activity of Digestarom^®^ that can be ascribable to its active compounds including anethole, carvacrol, limonene and thymol [14, 15]. However, other authors reported that these substances can modulate the immune responses and intestinal microbiota in rainbow trout [16]. Moreover, the inclusion of PHYTO^®^ in the diet reduced the susceptibility of European sea bass (*Dicentrarchus labrax*) to *Vibrio anguillarum* [17], which could be related to the antibacterial properties of its active compounds from garlic and labiatae plant extracts.

In conclusion, this study highlighted that Biocitro^®^ could be proposed as alternative to antibiotics for the treatment of fish diseases, being effective in increasing rainbow trout survival to lactococcosis probably by a direct antibacterial activity when administered as feed additive. However, further studies are needed to elucidate the exact mechanisms of its action and investigate whether Biocitro^®^ is also capable to enhance the immune response and/or induces changes in fish intestinal microbiota.

## Data Availability

The datasets used and/or analyzed during the current study are available from the corresponding author on reasonable request.
